# On the validity of “*Candidatus* Dirofilaria hongkongensis” and on the use of the provisional status *Candidatus* in zoological nomenclature

**DOI:** 10.1186/s13071-020-04158-3

**Published:** 2020-06-05

**Authors:** Filipe Dantas-Torres, Domenico Otranto

**Affiliations:** 1 Department of Immunology, Aggeu Magalhães Institute, Oswaldo Cruz Foundation (Fiocruz), Recife, Brazil; 2 Department of Veterinary Medicine, Università degli Studi di Bari, Valenzano, Bari, Italy; 3 Faculty of Veterinary Sciences, Bu-Ali Sina University, Hamedan, Iran

**Keywords:** *Dirofilaria*, Phylogeny, Taxonomy, New species, Nomenclature, Rules

## Abstract

The fast development of molecular taxonomy is impacting our knowledge of the world parasite diversity at an unprecedented level. A number of operational taxonomic units have been uncovered and new species described. However, it is not always that new parasite species are being described in compliance with the International Code of Zoological Nomenclature. This is the case of “*Candidatus* Dirofilaria hongkongensis”, a nematode found in dogs, jackals and humans in Hong Kong and parts of India. This name has been proposed without a formal description and without the designation of a holotype, and therefore is an unavailable name. Finally, we argue that using the provisional status *Candidatus* in zoological nomenclature is inappropriate, considering this term is not considered in the International Code of Zoological Nomenclature.

## Letter to the editor

*Dirofilaria immitis* and *Dirofilaria repens* are widespread nematodes of major medical and veterinary importance. *Dirofilaria immitis* is ubiquitous in distribution and *D. repens* is present in the Old World [1, 2]. They are transmitted to animals and humans *via* the bite of infected female mosquitoes belonging to numerous species around the world [1].

In 2012, To et al. [3] reported three human cases of dirofilariosis in Hong Kong. One patient presented with cervical lymphadenopathy, one with an abdominal subcutaneous mass, and the other with a subconjunctival nodule. Cytochrome *c* oxidase subunit 1 (*cox*1) gene sequences obtained from the three human patients were identical. Further analysis showed homologies of 96.2% and 89.3% to the *cox*1 gene of *D. repens* and *D. immitis*, respectively. In a similar manner, a sequence of the *18S*-ITS1-*5.8S* gene cluster was obtained from an intact worm, showing homologies of 94.0% and 94.9% to those of *D. repens* and *D. immitis*, respectively. To et al. [3] investigated the presence of this *Dirofilaria* sp. in dogs and cats, detecting 3% (6/200) of positive dogs and no positive cat. Finally, the *cox*1 gene and *18S*-ITS1-*5.8S* gene cluster obtained from dogs were found to be identical to those detected in the human patients. With solid evidence, To et al. [3] elegantly demonstrated that a new zoonotic species of the genus *Dirofilaria*, related to *D. repens*, was circulating among dogs and humans in Hong Kong, proposing the name “*Candidatus* Dirofilaria hongkongensis”.

Subsequently, this parasite has been referred to as *Dirofilaria hongkongensis*, *Candidatus* Dirofilaria hongkongensis, *Dirofilaria* sp. *hongkongensis*, *Dirofilaria* sp. “*hongkongensis*”, and *Candidatus* Dirofilaria (Nochtiella) Honkongensis, by different authors [3–15]. Unfortunately, To et al. [3] in the original reference to “*Candidatus* Dirofilaria hongkongensis” failed to identify either a holotype or give an appropriate morphological description, which are part of the rules of the International Code of Zoological Nomenclature (ICZN) [16]. Therefore, *Dirofilaria hongkongensis* must be considered *nomen nudum* and an unavailable name. This species name is not currently registered in ZooBank (as of 3 June 2020).

The parasite reported by To et al. [3] as “*Candidatus* Dirofilaria hongkongensis” has been detected in an Austrian traveller returning from the Indian subcontinent [9], in dogs, jackals and humans in south India [12, 13], and again in a human in Hong Kong [5, 15]. Other authors identified additional genotypes related to *D. repens*, referred to as *Candidatus* Dirofilaria sp. ‘Thailand II’ (also referred to as *Dirofilaria* sp. ‘Thailand II’) and *Dirofilaria* sp. “Thailand III” – in cats in Thailand [10, 11]. These genotypes were related but different from each other and different from “*Candidatus* Dirofilaria hongkongensis”.

Different genotypes related to *D. repens* or to *D. immitis* have been reported in the Americas [1]. For instance, a nematode extracted from the eye of a human patient in Pará State (northern Brazil), morphologically similar to, but genetically distinct from *D. immitis* (percentage of nucleotide difference 5% and 6% for *12S* rDNA and *cox*1, respectively) [17]. Such a high nucleotide variation suggested the existence of a cryptic species of *D*. *immitis* in Brazil or of a closely related species. Nonetheless, in the absence of more data (e.g. additional male and female specimens, but also microfilariae from a proper host), no new species name was proposed.

Incidentally, other authors have also been using the provisional status *Candidatus* in zoological nomenclature (e.g. *Candidatus* Babesia vesperuginis and *Candidatus* “Theileria senegalensis”) in recent years (e.g. [18, 19]). This category has been proposed by Murray & Schleifer [20] for recording the properties of putative taxa of prokaryotes and has been implemented by the International Committee on Systematics of Prokaryotes [21]. According to the International Code of Nomenclature of Prokaryotes (ICNP) [22], the provisional status *Candidatus* may be used to record the properties of putative taxa of prokaryotes and should be used for describing prokaryotic entities for which more than a mere nucleic acid sequence is available but for which characteristics required for description according to the ICNP are lacking. Furthermore, the ICNP provides a list of information that should be included in the description of a *Candidatus*: “(a) Genomic information, i.e. nucleic acid sequences apt to determine the phylogenetic position of the organism. (b) All information so far available on (c) structure and morphology (appropriate illustration) (d) physiology and metabolism (e) reproductive features (f) the natural environment, in which the organism can be identified by in situ hybridization or similar techniques for cell identification. (g) Any other available and suitable information” [22].

On the other hand, the provisional status *Candidatus* is not considered in the ICZN, which makes no reference regarding the use of this or similar term [16]. Therefore, the current use of this term in zoological nomenclature should be avoided, so as to avoid that several newly proposed names become unavailable. As emphasized elsewhere, the use of informal clade names is necessary until formal valid descriptions are available [23].

## Data Availability

Not applicable.

## References

[1] Dantas-Torres F, Otranto D (2020). Overview on *Dirofilaria immitis* in the Americas, with notes on other filarial worms infecting dogs. Vet Parasitol..

[2] Genchi C, Kramer LH (2020). The prevalence of *Dirofilaria immitis* and *D. repens* in the Old World. Vet Parasitol..

[3] To KK, Wong SS, Poon RW, Trendell-Smith NJ, Ngan AH, Lam JW (2012). A novel *Dirofilaria* species causing human and canine infections in Hong Kong. J Clin Microbiol..

[4] Suzuki J, Kobayashi S, Okata U, Matsuzaki H, Mori M, Chen KR (2015). Molecular analysis of *Dirofilaria repens* removed from a subcutaneous nodule in a Japanese woman after a tour to Europe. Parasite..

[5] Kwok RP, Chow PP, Lam JK, Fok AC, Jhanji V, Wong VW (2016). Human ocular dirofilariasis in Hong Kong. Optom Vis Sci..

[6] Liesner JM, Krücken J, Schaper R, Pachnicke S, Kohn B, Müller E (2016). Vector-borne pathogens in dogs and red foxes from the federal state of Brandenburg, Germany. Vet Parasitol..

[7] Rakova VM (2016). Dirofilariasis: current aspects of studies. Med Parazitol (Moscow)..

[8] Nazar N, Lakshmanan B, Jayavardhanan KK (2017). Molecular characterization of human *Dirofilaria* isolates from Kerala. Indian J Med Res..

[9] Winkler S, Pollreisz A, Georgopoulos M, Bagò-Horvath Z, Auer H, To KK (2017). *Candidatus* Dirofilaria hongkongensis as causative agent of human ocular filariosis after travel to India. Emerg Infect Dis..

[10] Yilmaz E, Fritzenwanker M, Pantchev N, Lendner M, Wongkamchai S, Otranto D (2016). The mitochondrial genomes of the zoonotic canine filarial parasites *Dirofilaria* (*Nochtiella*) *repens* and *Candidatus* Dirofilaria (Nochtiella) Honkongensis provide evidence for presence of cryptic species. PLoS Negl Trop Dis..

[11] Yilmaz E, Wongkamchai S, Ramünke S, Koutsovoulos GD, Blaxter ML, Poppert S (2019). High genetic diversity in the *Dirofilaria repens* species complex revealed by mitochondrial genomes of feline microfilaria samples from Narathiwat, Thailand. Transbound Emerg Dis..

[12] Gowrishankar S, Aravind M, Sastya S, Latha BR, Azhahianambi P, Vairamuthu S (2019). *Dirofilaria hongkongensis* - a first report of potential zoonotic dirofilariosis infection in dogs from Tamil Nadu. Vet Parasitol Reg Stud Reports..

[13] Pradeep RK, Nimisha M, Pakideery V, Johns J, Chandy G, Nair S (2019). Whether *Dirofilaria repens* parasites from South India belong to zoonotic *Candidatus* Dirofilaria hongkongensis (*Dirofilaria* sp. hongkongensis)?. Infect Genet Evol..

[14] Manoj RRS, Iatta R, Latrofa MS, Capozzi L, Raman M, Colella V (2020). Canine vector-borne pathogens from dogs and ticks from Tamil Nadu, India. Acta Trop..

[15] Xing F, Li X, Lo SKF, Poon RWS, Lau SKP, Woo PCY (2020). *Dirofilaria hongkongensis* infection presenting as recurrent shoulder mass. Parasitol Int..

[16] ICZN (1999). International Code of Zoological Nomenclature.

[17] Otranto D, Diniz DG, Dantas-Torres F, Casiraghi M, Almeida INF, Almeida LNF (2011). Human intraocular filariasis caused by *Dirofilaria* sp. nematode. Brazil. Emerg Infect Dis..

[18] Socolovschi C, Kernif T, Raoult D, Parola P (2012). *Borrelia*, *Rickettsia*, and *Ehrlichia* species in bat ticks, France, 2010. Emerg Infect Dis..

[19] Dahmana H, Granjon L, Diagne C, Davoust B, Fenollar F, Mediannikov O (2020). Rodents as hosts of pathogens and related zoonotic disease risk. Pathogens..

[20] Murray RG, Schleifer KH (1994). Taxonomic notes: a proposal for recording the properties of putative taxa of procaryotes. Int J Syst Bacteriol..

[21] Murray RG, Stackebrandt E (1995). Taxonomic note: implementation of the provisional status *Candidatus* for incompletely described procaryotes. Int J Syst Bacteriol..

[22] Parker CT, Tindall BJ, Garrity GM (2019). International code of nomenclature of prokaryotes. Int J Syst Evol Microbiol.

[23] Harris DJ (2016). Naming no names: comments on the taxonomy of small piroplasmids in canids. Parasit Vectors..

